# Risk Factors for Pediatric Invasive Group A Streptococcal Disease

**DOI:** 10.3201/eid1107.040900

**Published:** 2005-07

**Authors:** Stephanie H. Factor, Orin S. Levine, Lee H. Harrison, Monica M. Farley, Allison McGeer, Tami Skoff, Carolyn Wright, Benjamin Schwartz, Anne Schuchat

**Affiliations:** * Centers for Disease Control and Prevention, Atlanta, Georgia, USA;; † New York City Department of Health and Mental Hygiene, New York, New York, USA;; ‡ Johns Hopkins University, Baltimore, Maryland, USA;; § Emory University School of Medicine and the VA Medical Center, Atlanta, Georgia, USA;; ¶ Mount Sinai Hospital, Toronto, Ontario, Canada

**Keywords:** case-control studies, risk factors, Streptococcus pyogenes, toxic shock syndrome, streptococcus group A, matched case-control studies, necrotizing fasciitis

## Abstract

Invasive group A *Streptococcus* (GAS) infections can be fatal and can occur in healthy children. A case-control study identified factors associated with pediatric disease. Case-patients were identified when *Streptococcus pyogenes* was isolated from a normally sterile site, and matched controls (≥2) were identified by using sequential-digit dialing. All participants were noninstitutionalized surveillance-area residents <18 years of age. Conditional regression identified factors associated with invasive disease: other children living in the home (odds ratio [OR] = 16.85, p = 0.0002) and new use of nonsteroidal antiinflammatory drugs (OR = 10.64, p = 0.005) were associated with increased risk. More rooms in the home (OR = 0.67, p = 0.03) and household member(s) with runny nose (OR = 0.09, p = 0.002) were associated with decreased risk. Among children, household-level characteristics that influence exposure to GAS most affect development of invasive disease.

Invasive group A *Streptococcus* (GAS) infections include sepsis, bacteremic pneumonia, and dramatic, rapidly progressive syndromes such as necrotizing fasciitis and streptococcal toxic shock syndrome (STSS). An estimated 9,100 cases and 1,350 deaths occur in the United States each year, many of these among previously healthy children (1).

Previous studies identified associations between individual-level risk factors and pediatric invasive GAS disease. Many hospital-based case series (2–13) and 2 population-based studies (14,15) have associated varicella-zoster virus (VZV) infection with an increased risk for invasive GAS disease. Use of nonsteroidal antiinflammatory drugs (NSAIDs) was also associated (9,12), but whether NSAID use predisposed to or increased the severity of GAS infection (16) or was a marker of disease severity (17) is unclear. These previous studies were limited by the completeness of the data available from medical and laboratory records.

Household-level risk factors appear to play a role in disease development. Studies conducted in the 1950s demonstrated that school-age children were most often responsible for introducing a GAS strain into a household (18). Household transmission of GAS infection facilitated a communitywide GAS outbreak in southeastern Minnesota (19). Household crowding, measured by the number of persons in the home, and exposure to children with a sore throat in the home have been associated with increased risk for invasive GAS in adults (20). Household exposures have not been studied concomitantly with individual-level risk factors in children.

We conducted a population-based, case-control study with parental interviews to concomitantly study and identify individual- and household-level risk factors for invasive pediatric GAS disease. Parents could provide more complete data than medical record review. Simultaneous study of both types of data could also assess the relative effects of individual and household characteristics.

## Methods

Invasive GAS disease was defined as the isolation of *Streptococcus pyogenes* from a normally sterile site (including blood, cerebrospinal fluid, pleural fluid, peritoneal fluid, pericardial fluid, joint fluid, surgical specimens, bone, and scrotal fluid) in a noninstitutionalized resident, <18 years of age, in a surveillance area. Children who had GAS isolated from a sterile site >48 hours after hospital admission were presumed to have a nosocomial infection (21) and were excluded.

Cases of invasive GAS disease were identified through active, laboratory-based surveillance in 3 areas: metropolitan Atlanta, Georgia, from July 1, 1997, to June 30, 1999; metropolitan Baltimore, Maryland, from July 1, 1997, through June 30, 1999; and the Toronto-Peel region, Ontario, Canada, from July 1, 1997, through December 31, 1997. The surveillance area population was estimated to include 9 million people (3.6 in metropolitan Atlanta, 2.4 in metropolitan Baltimore, and 3.0 in the Toronto-Peel region based on 1997 Bureau of Census estimates [22]). All acute-care hospitals and laboratories serving the residents of the surveillance area were contacted biweekly and audited semiannually to identify patients with invasive GAS disease. For each case of pediatric invasive GAS identified, the standardized Active Bacterial Core Surveillance case-report form was completed by reviewing the hospital medical record. This form is used for all organisms under surveillance and includes all laboratory and clinical data needed to fulfill the criteria for streptococcal toxic shock syndrome (STSS) (23) and other clinical syndromes.

The method used to reach case-patients and identify controls has been described elsewhere (20). Briefly, a "case algorithm" was used to contact persons infected with invasive GAS. For each case-patient identified, up to 15 separate telephone calls were made to contact the parent or guardian. To maximize the likelihood of contacting the patient, the telephone calls were made on 5 nonconsecutive days, including at least 1 weekend day, during each of 3 different time periods (8:00 a.m.–12:00 p.m., 12:01–5:00 p.m., 5:01–8:00 p.m.). Case-patients were eligible if their enrollment was complete within 3 months of onset of GAS disease. Family members of deceased case-patients were interviewed, and non–English-speaking patients were included if individual surveillance sites had the resources to communicate with the patients in their language.

A population-based sample of matched controls was selected through systematic, sequential-digit telephone dialing. Case-patients and controls were matched by age group, postal or zip code, and telephone exchange to control for age and socioeconomic status. Age groups were defined as 0–23 months, 24–59 months, and 5–17 years. Case-patients without telephones were excluded.

When experienced surveillance personnel reached the parent or guardian of a case-patient or control, they explained the purpose of the study, obtained informed consent, and administered a standardized questionnaire. The questionnaire included questions on demographics, socioeconomic status, age-specific activities like breastfeeding and preschool, medical history, and household characteristics. The household characteristics included physical space, number of persons in the household, persons who smoke, and symptoms in other persons in the past 2 weeks for controls or in the 2 weeks before invasive GAS disease among case-patients. Within the medical history section, we differentiated between "regular" NSAID use and "new" NSAID use. New NSAID use indicated that the case-patient had started using NSAIDs in the 2 weeks before illness was diagnosed or that a control participant had started using NSAIDs in the 2 weeks before the interview. The parent or guardian for case-patients and controls was allowed to self-define regular use of NSAIDS. This study was approved by the institutional review boards at the Centers for Disease Control and Prevention and at each surveillance site.

Odds ratios (ORs) for each potential risk factor were determined by using conditional logistic regression (Proc PHREG, SAS Version 6.12, SAS Institute Inc., Cary, NC, USA), controlling for sex and race. Variables with p<0.20 in individual analyses were included in multivariable analysis. Computer-assisted and manual forward, backward, and stepwise conditional logistic regression identified risk factors independently associated with invasive GAS disease. ORs with 95% confidence intervals (CIs) that did not include 1.00 and p values <0.05 were considered significant in multivariable analysis.

## Results

Surveillance identified 72 episodes of invasive GAS disease among children <18 years of age. Eight had nosocomial infection and were ineligible. Of the 64 remaining, 38 were enrolled, 5 parents or guardians refused to participate, 3 were not reached after exhausting the telephone call algorithm, and 18 did not participate for other reasons, including a time lapse of >3 months after the illness, incomplete or incorrect contact information (i.e., wrong phone number, disconnected phone, no phone, homelessness), and difficulty communicating over the phone (i.e., poor communication skills, non–English-speaking parent or guardian). No statistical differences in race, sex, age, or death rate were seen between enrolled and nonenrolled patients.

The number of case-patients enrolled varied by area: 20 (53%) from Atlanta, 11 (29%) from Baltimore, and 7 (18%) from Toronto. Ten (26%) patients were 0–23 months of age, 7 (18%) were 24–59 months, and 21 (55%) were 5–18 years. Forty-seven percent were boys. Of enrolled patients, 24 (63%) were white, 13 (34%) were African American, and 1 (3%) did not specify ethnicity. Two patients (5%) died of the disease; both were diagnosed with primary bacteremia without focus. Primary bacteremia and cellulitis were the 2 most common diagnoses (Table 1). No cases of STSS were found.

**Table 1:** Clinical syndromes of children with invasive group A streptococcal disease, Atlanta, Baltimore, and Toronto, 1997–1999*

Clinical syndrome	No. patients (%)
Primary bacteremia (without focus)	18 (47)
Cellulitis	6 (16)
Septic arthritis	4 (11)
Necrotizing fasciitis	1 (3)
Pneumonia	1 (3)
Otitis	1 (3)
Peritonitis	1 (3)
Abscess	1 (3)
Appendicitis	1 (3)

*Patients may appear in >1 category; data not available for all patients.

Several factors were associated with invasive GAS disease (Table 2). When sex and race were controlled for in individual analysis, having a primary caretaker who smokes, presence of ≥1 other children in the home, and new use of NSAIDs were associated with an increased risk for invasive GAS disease (p≤0.05); more rooms in the home, higher level of parental education, and a household member with a runny nose (rhinitis) in the past 2 weeks were associated with a decreased risk for invasive GAS disease (p≤0.05). By using multivariable conditional regression and controlling for sex and race, 4 risk factors were found to be independently associated with invasive GAS disease: having ≥1 other children in the home (OR = 16.85, p = 0.0002) and new use of NSAIDs (OR = 10.64, p = 0.005) were associated with an increased risk, and more rooms in the home (OR = 0.67, p = 0.03) and having a household member with a runny nose in the past 2 weeks (OR = 0.09, p = 0.002) were associated with a decreased risk.

**Table 2:** Individual and multivariable analysis for risk factors for invasive group A streptococcal disease among case-patients and controls*

Variable	Case-patients (N = 38) (%)	Controls (N = 78) (%)	Individual analysis		Multivariable analysis	
			OR (95% CI)	p value	OR (95% CI)	p value
No. persons in home (mean)	4.42	4.02	1.28 (.91–1.78)†	0.15		
Live in single-family home	23 (62)	59 (76)	0.49 (0.21–1.16)	0.11		
No. rooms in home (mean)	6.39	7.27	0.81 (0.66–0.99)‡	0.04	0.67 (0.51–0.88)‡	0.03
No. smokers in home						
0	21 (57)	53 (68)	1.43 (0.87–2.32)§	0.16		
1	9 (24)	16 (21)				
2	6 (16)	7 (9)				
3	1 (3)	2 (3)				
Primary caretaker is a smoker	11 (30)	13 (17)	2.71 (1.02–7.21)	.05		
≥1 other child <18 years living in the home	33 (87)	43 (56)	5.76 (1.95–16.96)	.002	16.85 (3.90–72.84)	0.0002
≥1 other person in the household with a cough	4 (11)	17 (22)	0.40 (0.12–1.32)	0.13		
≥1 other person in the household with a runny nose	4 (11)	26 (34)	0.25 (0.08–0.80)	0.02	0.09 (0.01–0.40)	0.002
HIV-positive	1 (3)	0	Undefined	0.99		
Eczema	8 (21)	16 (21)	0.99 (0.38–2.64)	0.99		
Varicella-zoster virus infection	3 (8)	0	Undefined	0.99		
Vaccinated with varicella-zoster virus vaccine	12 (46)	26 (47)	0.93 (0.36–2.40)	0.88		
New use of NSAIDs	9 (24)	7 (9)	3.15 (1.07–9.29)	0.04	10.64 (2.08–54.61)	0.005
Use of corticosteroids	1 (3)	2 (3)	0.93 (0.08–11.02)	0.95		
Parent/guardian education						
Some HS	4 (11)	3 (4)	0.69 (0.51–0.91)	0.01		
HS graduate or GED	12 (32)	13 (17)				
Technical school	2 (5)	5 (6)				
Some college	10 (26)	23 (29)				
College graduate	7 (18)	23 (29)				
Postgraduate study or professional	2 (5)	10 (13)				
Household income						
<$15,000	7 (21)	2 (3)	0.70 (0.48–1.01)	0.06		
$15,001–$30,000	8 (24)	11 (18)				
$30,001–$45,000	4 (12)	13 (22)				
$45,001–$60,000	6 (18)	17 (28)				
≥$60,001	9 (26)	17 (28)				
Ever breastfed¶	0	12 (71)	0 (Undefined)	1.00		

*Data were not available for all variables for all case-patients and controls. Analyses controlled for race and sex. OR, odds ratio; CI, confidence interval; NSAID, nonsteroidal antiinflammatory drug; HS, high school; GED, general equivalency diploma. †Increase in risk with each additional person living in home. Persons living in the home were evaluated as a continuous variable in conditional logistic regression. ‡Decrease in risk with each additional room in home. Rooms were evaluated as a continuous variable in conditional logistic regression. §Increase in risk with each additional smoker in home. Smokers in the home were evaluated as a continuous variable in conditional logistic regression. ¶Question asked only of children <2 years of age; 8 case-patients and 17 controls were <2 years. Because none of the case-patients were breastfed, the calculated OR = 0, and the confidence interval is not defined.

VZV and HIV infection occurred only in case-patients (n = 3 and n = 1, respectively). Patients and controls were equally likely to be vaccinated against VZV (OR = 0.93, p = 0.88). Of the 3 case-patients with history of VZV infection, 1 reported new use of NSAIDs. Among participants 0–23 months of age, 12 (71%) of 17 controls were currently being or had ever been breastfed compared to 0 of 8 case-patients (OR = 0, p = 1.00).

## Discussion

This study suggests that children bring GAS into the home and that crowding, measured by the number of rooms in the home, influences the development of invasive GAS disease. The protective association of rhinitis was unexpected, and the mechanism of protection is not obvious. Individual-level risk factors seem to play a less important role. Although NSAID use is associated with invasive disease, the measurements of new use and regular use are too crude to clearly identify their role as a risk factor.

The roles of children and crowding are expected and have been suggested by previous studies. Children are most likely to introduce GAS infection into the home (18), children spread GAS in the home (19), and children with sore throats are likely reservoirs of GAS for adults who develop invasive disease (20). Crowding, measured by number of people in the home, increases risk for acquiring disease among adults >45 years of age (20).

Although the association between rhinitis and invasive GAS infection may be spurious, data support a true relationship. Among persons with sore throats, those with rhinitis are less likely to have GAS pharyngitis than are those without rhinitis (24,25). This finding suggests that controls were less likely to be exposed to persons with GAS pharyngitis than were case-patients.

Although only significant in individual analysis in this study, cigarette smoke has been independently associated with other invasive bacterial infections in other studies. Increased risk for invasive meningococcal disease in children <18 years is associated with having a mother who smokes (26), and increased risk for invasive pneumococcal disease in immunocompetent, nonelderly adults is associated with both being a smoker and being exposed to other smokers (27). Larger numbers of case-patients may show an association.

A large difference was seen in the proportions of patients and controls who have been breastfed, although this difference was not significant. Breastfeeding may protect against invasive GAS disease as it does against other invasive bacterial diseases. Previous studies found current breastfeeding protective against invasive pneumococcal disease in children 2–11 months of age (28) and against invasive *Haemophilus influenzae* type B disease in children <6 months of age (29). Data suggest that in addition to containing protective antibodies against these organisms, breast milk can inhibit bacterial colonization independent of antibody concentration (30). Although HIV infection is a risk factor for invasive GAS infection in adults (20,31) and VZV infection is a risk factor for invasive GAS infection in children (14–15), this study had too few patients to comment on either.

This study has several limitations. The small size limits the statistical power to identify associations between individual- and household-level characteristics and invasive GAS disease. Some questions were only asked of subgroups, further decreasing power to detect associations. Using sequential-digit dialing and matching on zip or postal codes controlled for socioeconomic and community-level risk factors. These factors could therefore not be studied. This method may limit the generalizability of the findings; the study population included only persons with phones and, specifically, persons likely to answer their phones.

The associations in this study all suggest that development of pediatric invasive GAS disease is largely related to opportunities for exposure to GAS, as measured by exposure to children, other persons, and persons with GAS infections. Individual-level risk factors in children are less important. Breastfeeding young children and nonsmoking by their household contacts may be preventive and should be encouraged.
